# Nano-Structured Ridged Micro-Filaments (≥100 µm Diameter) Produced Using a Single Step Strategy for Improved Bone Cell Adhesion and Proliferation in Textile Scaffolds

**DOI:** 10.3390/molecules27123790

**Published:** 2022-06-13

**Authors:** Nemeshwaree Behary, Sandy Eap, Aurélie Cayla, Feng Chai, Nadia Benkirane-Jessel, Christine Campagne

**Affiliations:** 1 ENSAIT, ULR 2461—GEMTEX—Génie et Matériaux Textiles, University of Lille, F-59000 Lille, France; nmassika.behary@ensait.fr (N.B.); christine.campagne@ensait.fr (C.C.); 2 INSERM-UNISTRA UMR 1260, Regenerative Nanomedicine RNM, FMTS, 1 rue Eugène Boeckel, F-67084 Strasbourg, France; sandy.eap@inserm.fr (S.E.); nadia.jessel@inserm.fr (N.B.-J.); 3 INSERM, U1008-Controlled Drug Delivery Systems and Biomaterials, University of Lille, CHU Lille, F-59000 Lille, France; fchai@univ-lille.fr

**Keywords:** microfilaments, PLLA, nano-ridged fiber surface, melt-spinning, bone cell engineering, osteogenic expression

## Abstract

Textile scaffolds that are either 2D or 3D with tunable shapes and pore sizes can be made through textile processing (weaving, knitting, braiding, nonwovens) using microfilaments. However, these filaments lack nano-topographical features to improve bone cell adhesion and proliferation. Moreover, the diameter of such filaments should be higher than that used for classical textiles (10–30 µm) to enable adhesion and the efficient spreading of the osteoblast cell (>30 µm diameter). We report, for the first time, the fabrication of biodegradable nanostructured cylindrical PLLA (poly-L-Lactic acid) microfilaments of diameters 100 µm and 230 µm, using a single step melt-spinning process for straightforward integration of nano-scale ridge-like structures oriented in the fiber length direction. Appropriate drawing speed and temperature used during the filament spinning allowed for the creation of instabilities giving rise to nanofibrillar ridges, as observed by AFM (Atomic Force Microscopy). These micro-filaments were hydrophobic, and had reduced crystallinity and mechanical strength, but could still be processed into 2D/3D textile scaffolds of various shapes. Biological tests carried out on the woven scaffolds made from these nano-structured micro filaments showed excellent human bone cell MG 63 adhesion and proliferation, better than on smooth 30 µm- diameter fibers. Elongated filopodia of the osteoblast, intimately anchored to the nano-structured filaments, was observed. The filaments also induced in vitro osteogenic expression, as shown by the expression of osteocalcin and bone sialoprotein after 21 days of culture. This work deals with the fabrication of a new generation of nano-structured micro-filament for use as scaffolds of different shapes suited for bone cell engineering.

## 1. Introduction

Research in Bone Tissue Engineering is necessary for the healing of some bone diseases and for the regeneration of bone defects [1], as well as for the treatment of age-related orthopedic disorders [2,3,4,5]. Bone Tissue Engineering scaffolds need to be constructed in various shapes: “two-dimensional”, i.e., interacting with cells only at the implant surface, and “three-dimensional”, allowing the ingrowth of the bone tissue inside the implant [6]. Current 3D printing or bio-printing [7] allows for the processing of various shaped scaffolds with controllable pore sizes, which have promising results in the building of many tissues [8,9]. There are, however, many challenges that still need to be addressed in the field of bone tissue engineering: hydrogel or ceramic-based scaffolds are of low strength while the brittleness of ceramics makes them difficult to handle during processing and use [9,10]. Textile scaffolds based on biodegradable polymeric fibers can be a precious alternative, since the interlacing or interlocking of the microfilaments, in 2D/3D woven, knitted, or braided textiles, leads to improved mechanical tensile and flexural strength, leading to their use in such fields as aerospace [11]. Such textile structures equally provide scaffolds of tunable shapes and pore sizes [12]. However, the microfilaments lack the nano-topographical features necessary for bone cell adhesion and proliferation. Indeed, in addition to the degradability of the scaffold, surface energy, and charge, the physical surface properties of a biomaterial–surface roughness and topography–determine the amount and the quality of cell adherence [4,5,6,13,14]. Nano-structured surfaces preferentially adsorb vitronectin ECM (due to its relatively small, linear, and non-complicated molecules), and this protein is preferentially recognized by osteoblasts over other cell types [5,15,16]. Creating nanostructures at the biomaterial surface seems thus to be the most efficient approach for bone cell adhesion and colonization [5,6,13,14].

Several researches have shown that surfaces of roughness in tens of nanometers are preferred by bone cells for adhesion, growth, differentiation, and phenotypic maturation, rather than flat surfaces and surfaces with submicron or micro-scale roughness [5,6,15,16]. Nanotexturing of titanium-based surfaces upregulates the expression of bone sialoprotein and osteopontin by cultured osteogenic cells [5,17]. Microscale irregularities, on the other hand, can hamper cell spreading and thus may slow down cell proliferation [18,19].

**Figure 1:**
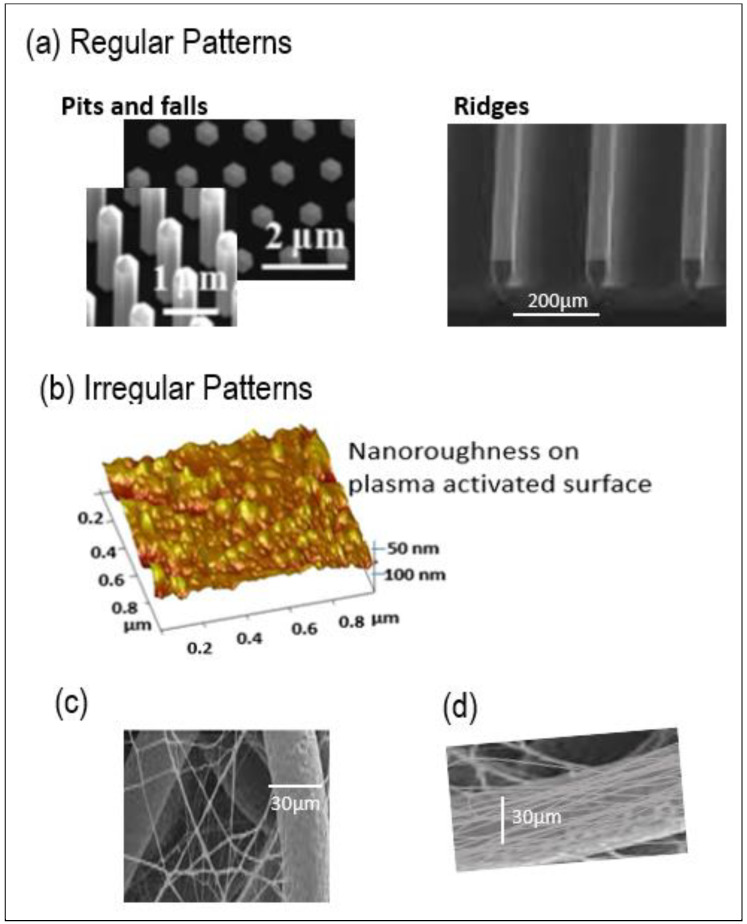
Nanostructures can be in the form of (**a**) Regular patterns (regular falls, pits-cavity or ridges); (**b**) Irregular patterns and (**c**,**d**) Microfibers of nonwovens can be nanostructured using nanofibers.

In addition to the dimension of surface roughness, surface topography and patterning also has an influence on cell-material interaction: important parameters are not only the size of the surface irregularities, but also their shape and distribution (regular or irregular distribution) on the surface of the material [5]. In literature, such nanostructures (short range order) which can be obtained by different techniques, can be in the form of regular patterns (regular falls, pits-cavity, or ridges) or randomly (irregular) disposed nanometric patterns at surfaces (Figure 1a,b) [20]. Webster et al. [21] found that the highest numbers of initially adhering rat osteoblast cells were found on TiO_2_ with grain size of 20–32 nm and Al_2_O_3_ with grains of 20–49 nm. Kalbáčová et al. [22] showed that nanocrystalline diamond film surfaces with RMS of 20 nm provided the best support for the initial adhesion of human osteoblast cells. Vandrovcová et al. [6] showed that on Ti O_2_ or glass, a nano-roughness of Ra = 40 nm induced a larger human osteoblast like the MG 63 cell spreading area and significantly higher cell numbers on day 7 than a surface roughness of Ra of 100 and 170 nm. De Oliveira [17] showed that osteoblasts grew on the nanotextured surfaces, which contained nano-pits with a honeycomb-like appearance and were in the 10 nm range. Moreover, the alignment of osteoblast cells along the grooves, steps, and ridges were reported. Lenhert et al. [23] showed that grooves of 50–150 nm depth and with a periodicity of 500 nm promoted the alignment of osteoblasts along the grooves on polystyrene polymer.

Different micromachining and microfabrication techniques generate the nanoscale topography in regular or irregular patterns on synthetic surfaces [20]. Soft lithography and nano-transfer molding generate regular patterns. Reactive ion etching, different nanomaterial fabrication methods, and SGDR (spontaneous galvanic displacement reaction) allows to obtain irregular patterns [23].

As far as fibrous textile structures are concerned, electro-spun webs consisting of nanofibers are ideal in nano surface roughness to promote initial cell adhesion [24] but, cell infiltration inside the nanofibrous scaffold is limited because of the nano-sized pores provided by the electro-spun webs [25,26]. Pore size ranges from 100 to 600 μm [27,28] have been suggested for improved bone cell infiltration and nutriment diffusion and this can be provided by micro-fibrous 2D-3D scaffolds which have improved mechanical properties. However, microfibers lack nano-topographical features, and cell proliferation in 3D microfibrous scaffolds is slow because fewer cells are directly attached to the micro-sized fiber surfaces [29]. Moreover, cells on microfibers have been found to be more spherical than those cultured on flat or nanostructured surfaces, due to a reduction in the surface cell spreading [30].

Different methods have been studied to introduce nanofeatures into microfibrous scaffolds [31], to improve cell adhesion and proliferation. Previous works report surface etching using chemical or plasma methods to introduce nano-topographies at the microfiber surface, which is readily subjected to ageing [32]. Nanoparticles such as HA-hydroxyapatite [33] and CNT-carbon nanotubes [34], as well as nanofibers, have been used to produce nanofeatures on micro-sized scaffolds, using the sol–gel process [35], polymer coating, or electrospinning [36,37], and the Layer-by-Layer deposition technique described in our previous work [38]. Impregnation of nanofibers on microfiber-based nonwovens, lead to nanofiber deposition in the internal pores of the scaffold too (Figure 1c), which limits cell migration inside the scaffold [37]. Electrospun nanofibers can be homogeneously deposited onto individual microfibers (Figure 1d) using sequential electrospinning of nanofibers on top of microfibers [31] and then the nanofiber-coated microfibers can be made into scaffolds with the desirable shapes and tunable porosity. Cellular infiltration and spreading along the nanofiber-coated microfibers were observed in the scaffolds, which also maintained their surface and structural properties. However, the processing of such microfibers into traditional textiles such as 2D or 3D woven, or knitted textiles may pose a problem: friction between the nanofiber coated microfilaments and the textile processing machine elements, such as knitting needles and weaving loom eye wire heddles, causes the partial removal of the adhered nanofiber coating.

Many biopolymers, such as polysaccharides, have been used for cell culture [31,39], however, since bone cell adhesion is mainly influenced by surface nano-topographies, our team, specialized in fiber melt-spinning, worked on determining the parameters for directly integrating the nanoscale ridge-shape nano-scale topography on micro-scale biodegradable PLLA (poly-L-Lactide acid) filaments, on the basis of previous research works [40,41,42,43] which have shown that, during melt-spinning of filaments, there can be transition from a smooth surface to a nearly periodic ridge-like fiber surface, depending on the spinning parameters used. The most frequently observed defects, such as sharkskin [44], occur in the fiber radial axis. However, other types of surface defects having a fibrillar morphology oriented in the fiber length direction [45] have also been observed on melt-spun PLA [40,41] and polyamide-6 fiber surfaces [46]. While most research works address the suppression of surface defects to produce fibers with smooth surface suited to textile processing, the aim of the present work was to induce instabilities during the melt-spinning, leading to the nano-structuration of a microfiber surface, and creating an appropriate ridge-shaped nanostructured fiber surface for improved osteoblast cell adhesion.

This work describes for the first time the use of melt spinning conditions’ monitoring to confer nanostructured-ridged surface to PLA fibers for improved osteoblast cell adhesion and proliferation. Use of nano-structured microfiber (>100 µm) can help to improve the spreading of 30 µm size osteoblasts, which usually form numerous filopodia extensions when adhering to surfaces. Hence, filaments of diameter 30 µm, 100 µm and 230 µm were spun.

Topographical analysis was carried out by AFM (atomic force microscopy) to detect any fiber surface nano-structuration. Individual fiber tensile strength, as well as the thermal properties of each filament, were determined. Textile processing assays were performed to test the processability of the microfilaments into knitted, braided, and woven 2D and 3D structures, with tunable pore sizes. The pore size of scaffolds was characterized by visual analysis using optical microscopy. Biological assays were carried with Human MG 63 osteoblast cells on scaffolds made from the three different filaments. Cell proliferation up to 21 days was determined using cell viability test with AlamarBlue^®^. Immunofluorescence staining was also carried out to observe cell nuclei and actin as well as osteogenic markers such as Osteocalcin and Bone sialoprotein (BSPII).

## 2. Materials and Methods

### 2.1. Materials

PLA-Polylactic acid (NatureWorks^®^ 6202D) was purchased from Cargill–Dow (Vilvoorde, Belgium) with the following characteristics: Mw = 97,000 g/mol; Tg = 58 °C; Tf = 168 °C; and D-isomer = 2% and L-isomer = 98%. The grade of the processed polymer was chosen for their spinnability by melt process with a Melt Flow Index of 32 g/10 min at 230 °C, 2.16 kg.

### 2.2. Spinning of Micro/Micro-Sized Filaments (or Fibers)

We successfully used a single-step melt-spinning process fabrication method for producing biodegradable PLLA (L-Polylactic acid) nanostructured micro/microfibers.

**Figure 2:**
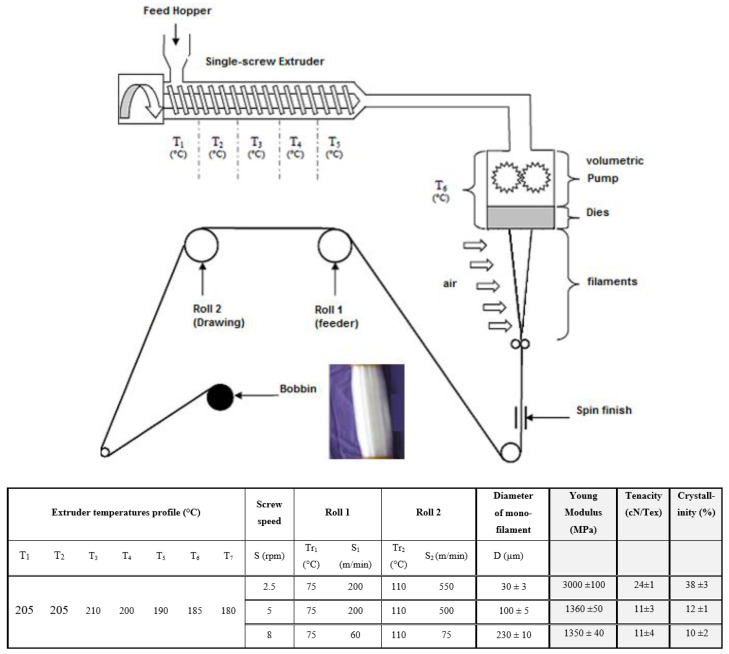
Melt–spin process diagram. The parameters of melt–spin to obtain PLLA filaments of various diameters are given in the table. In grey shading, data for the physical and mechanical properties of each filament are given.

Spinning conditions to produce smooth PLLA cylindrical smooth fibers using a melt spinning machine (driver SPINBOY I, Busschaert Engineering, Deerlijk, Belgium) were already described in previous research work carried out in our laboratory [47]. Dried PLLA pellets NatureWorks^®^ 6202D were fed in the hopper, passed through a heated single-screw extruder (with temperature gradient varying from 185 to 210 °C-Figure 2), while a volumetric pump ensured injection of the molten polymer with a constant rotation speed, thereby ensuring a constant flow of polymer into the die-bearing holes (Figure 2). Filaments emerging from the die head were cooled in air, gathered to form a multifilament, which is rolled up on two heated rolls with varying speeds to ensure drawing of the filaments (Figure 2). In this study, in order to obtain PLLA micro/microfibers ranging from 30 µm to 230 µm in diameter, a die head with two holes (instead of a classical head with eighty holes) was used. To create instabilities during spinning, the screw speed (S) as well as the speeds of Roll 1 (S1) and Roll 2 (S2), were varied, while temperature gradient along the screw as well as the temperatures of Roll 1 and Roll 2 were kept constant (see Figure 2). The theoretical drawing of the filament is given by the draw ratio DR = S2/S1. The experimental parameters used to produce the three different micro/micro-sized filaments named FiPLA30, FiPLA100, and FiPLA230, of diameter 30 µm, 100 µm, and 230 µm, respectively, are detailed in the table illustrated in Figure 1. With the highest drawing ratio, the 30 µm diameter filament FiPLA30 was produced. The draw ratio was only one for the FiPLA230 filament.

The PLLA filament diameter was measured with observation of a cross section of each filament by a digital camera coupled with a binocular microscope. The diameter of at least 20 monofilaments was determined to obtain a representative average value (noted D, see table in Figure 2).

### 2.3. Processing of PLA Micro/Micro-Sized Fibres into Textile Scaffold

**Figure 3:**
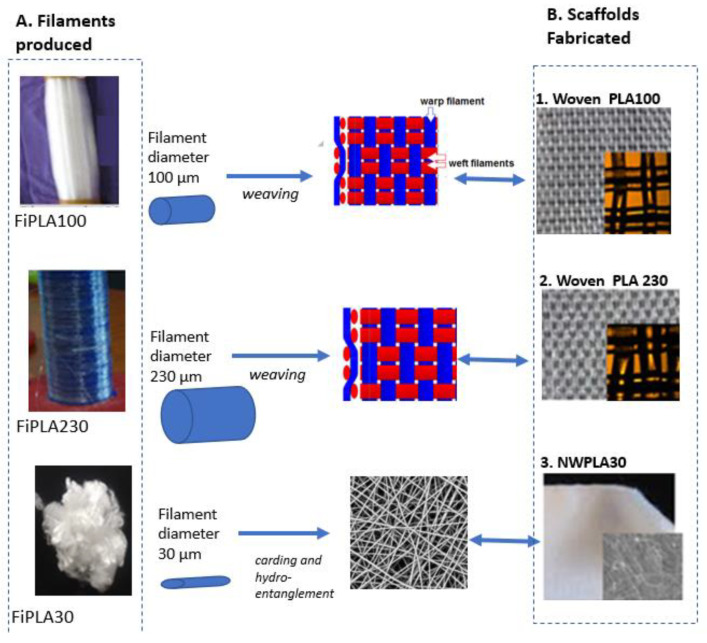
Morphological Analyses of PLLA filaments fabricated, and the fibrous PLLA scaffolds tested. (**A**). Photographs of the three different PLLA filaments of diameter 30, 100 and 230 µm; (**B**) Different scaffolds subjected to biological tests: two woven scaffolds WovenPLA100 and WovenPLA230 made from nanostructured PLLA filaments of 100 and 230 µm diameter, respectively, and a nonwoven scaffold NWPLA30 made from a smooth 30 µm diameter filament. In (**B**) on the front—pictures of scaffolds using binocular lenses are shown.

Weaving of the 100 or 230 µm diameter monofilaments was carried out to produce a 2D woven scaffold. A woven fabric is generally composed of longitudinal warp yarn interlaced at right angles with lateral weft yarns. Weaving of such filaments required technological modifications of the weaving loom Patronic B60 from ARM (Cambiriage, UK). The same filament was used as warp and weft. The representative weave pattern of the two woven scaffolds are shown in Figure 3B. Only the 100 µm and 230 µm-diameter PLLA monofilaments were processed into simple porous woven structures, denominated WovenPLA100 and Woven PLA230, respectively. The 30 µm-diameter PLA fibers could not be woven because of their fineness, and were thus converted into a nonwoven web-NWPLA30 (see Figure 3B), using carding and hydro-entanglement as described in a previous paper (Houdali, 2017).

### 2.4. Cleaning of Wovens and Nonwoven to Remove Spin Finish

Spin finish is added onto the filaments to avoid their breakage during melt–spinning and further processing. Without removal of this spin finish, no cell adhesion and cell growth occurred. Woven and nonwoven samples were subjected to Soxhlet washing, successively with petrol ether and ethanol separately, for 2 h. After each washing, the samples were dried at room temperature for 24 h to evaporate all of the solvent. Then, they were subjected to rinsing another three successive times in pure sterilized water at room temperature (during 15 min) using ultrasound. The surface tension of the last rinse water measured was 72.3 mN/m, which is the surface tension of pure water. This confirmed the removal of all spin finish impurities from the PLA fiber surface.

Samples were sterilized in ethanol for 5 min before biological characterization.

### 2.5. Characterization Methods

Fiber surface topography was studied by Atomic Force microscopy, and the fiber surface water contact angle was also measured. Additionally, the mechanical and thermal behavior of the fibers, were characterized to ensure their processability into textile structures using traditional processing (weaving, knitting, braiding machines).

#### 2.5.1. Fiber Surface Properties

- Atomic Force Microscopy (AFM) analysis

Investigation using a “Nanoscope III” from Digital Instrument (UK) was carried out for AFM imaging in the Tapping mode. Tapping mode tips ‘Budget sensor’ from ‘Nanoandmore’, of length 125 µm, made of a monolithic Silicon probe with Aluminum reflex coating and with a resonance frequency of 300 kHz, were used. Tapping mode imaging was carried out in ambient air. AFM imaging was carried out on five different fiber samples selected randomly. Roughness parameters, Ra and Rq, were calculated for each scanned surface area, as described in our past paper [48].

- Water Contact angle

For measuring the water contact angle greater than 90°, the sessile drop method using “Digidrop” from GBX Instrument (Drome, France) was used. However, for lower contact angles (<90°), the water drop was absorbed by the porous woven structure. So, a more precise method, developed in several of our previous works, called the wicking test was performed to calculate the water contact angle. A rectangular piece of woven or nonwoven PLA was connected to a tensiometer at the weighing position, and was brought into contact with the water placed in a container. On immediate contact, a meniscus weight (Wm) was measured, and the water contact angle determined from this meniscus weight [49].

- Tensile strength characteristics of PLA monofilaments

The measurements of the mechanical properties of the PLA monofilaments were carried out following the standard NF EN ISO 5079. A mechanical tester system MTS 2/M associated with a strength sensor of 1 kN was used with special clips for yarns (“capstan grips”). All tests were carried out under standard atmosphere (temperature of (20 ± 2) °C and relative humidity of (65 ± 5)%).

#### 2.5.2. Thermal Characterizations of PLA Monofilaments

Crystallinity of monofilaments measured by Differential Scanning Calorimetry (DSC) Characterizations of each monofilament were performed on a 2920 Modulated DSC (TA Instruments, New Castle, DE, USA) with typically 5 mg ± 0.1 mg of dry material. The DSC analysis was carried out under nitrogen atmosphere (with a flow of 50 mL/min) and samples were subjected to two identical heating cycles in the following manner: an increase from −20 °C to 200 °C at 10 K/min, followed by an isotherm at 200 °C during 5 min, and then a cooling at 10 °C/min down to 20 °C. was analyzed to determine the Melting enthalpy ($\Delta H_{m}^{}$) and temperature (T_m_), as well as the cold Crystallization enthalpy ($\Delta H_{cc}^{}$) and temperature (T_cc_). The crystallinity degree (χ) of PLA was calculated according to Equation (1):

(1) $$\chi (\%)=\frac{\Delta H_{m}-\Delta H_{cc}}{\Delta H_{m}^{0}}$$

where: is the reference enthalpy defined as the heat of a 100% crystalline sample. The thermodynamic enthalpy of crystallization used for the crystallinity calculation is $\Delta H_{m}^{0}(PLA)=$ 93.6 J·g^−1^.

### 2.6. Biological Tests

Biological assays were carried out with human MG 63 osteoblast cells, by the participation of two different biological research laboratories. Cell proliferation tests, carried out by INSERM U1008 (Lille, France), consisted in direct seeding of the MG 63 cells onto the PLA disks and evaluating the number of adhered cells after 3 and 6 days. Further biological assays were carried out by INSERM UMR 1109 (Strasbourg, France) by placing woven PLA discs in a cell culture medium already seeded with MG 63 osteoblast cells. Cell proliferation up to 21 days was followed and Immunofluorescence staining was carried out after 21 days

#### 2.6.1. MG 63 Osteoblastic Cell Proliferation Assays

Human osteoblast osteosarcoma MG63s were cultured in a specific osteoblast growth medium (DMEM (Dulbecco’s Modified Eagle’s Medium)) containing supplement mix 50 U/mL penicillin and 50 μg/mL streptomycin (Ozyme, Montigny-le-Bretonneux, France). The cells were incubated at 37 °C in a humidified atmosphere of 5% CO_2_. When cells reached sub-confluence, they were harvested with trypsin (Ozyme) and sub-cultured.

***(a)***  ***Seeding of MG 63 cells on the PLA woven, nonwoven and film discs (3 and 6 days of culture)***

Different PLA disk (Ø11 mm) samples were each placed in the bottom of 24-well cell culture plates (Costar^®^, Starlab) after sterilization with ethanol. 4 different PLA structures were tested: the two wovens (WovenPLA100 and WovenPLA230) made from the nano-structured filaments of diameter 100 µm and 230 µm, respectively), the nonwoven -NWPLA30 (made from the 30 µm diameter filament), and a 1-mm thick PLA film. Then, 6 × 10^3^ MG 63 osteoblastic cells were gently seeded in each well. The wells without disk sample only filled with cell suspension served as the positive control (tissue culture polystyrene, TCPS). The duration of cell culture was 3 and 6 days without renewal of the culture medium.

Evaluation of the number of adhered cells and cell vitality, after 3- and 6-day incubation, were carried out using a cell counter and AlamarBlue^®^, respectively. Briefly, the MG 63osteoblastic cells were detached by 0.05% trypsin–EDTA solution and then counted with a particle counter (Z1, Coulter Electronics Ltd., Luton, UK). Three assays were separately preformed and each assay was triplicate. The final results were rated as the mean of all assays.

AlamarBlue^®^ (Thermo Fisher Scientific, Waltham, MA, USA) was also used to assess cell vitality after 3 and 6 days of culture. Cells were incubated in 500 µL of 10% alamarBlue^®^/Dulbecco’s Modified Eagle’s Medium solution in a humidified atmosphere at 37 °C and 5% CO_2_. After 3 h, 150 μL of incubation media were transferred to 96-well plates and measured at 590 nm and 630 nm in order to determine the percentage of alamarBlue^®^ reduction. Results are expressed in terms of % with respect to the alamarBlue^®^ reduction obtained with the TCPS control sample. The statistical analyses were obtained by the two way ANOVA test in Excel 2011, Microsoft Office (Microsoft Corporation, Redmond, WA, USA).

***(b)***  ***Seeding of MG 63 cells under the woven PLA discs (up to 21 days of culture)***

A 48-well cell culture plate was used. A total of 4 × 10^3^ MG 63 cells were seeded in each well before placing the PLA discs (Ø 11 mm). Only the two wovens, WovenPLA100 and Woven PLA230, were tested. The culture medium was changed every 3 days. After 3, 14, and 21 days of culture, cell proliferation was determined by using 10% alamarBlue^®^, as described above. Results are presented in the terms of % of alamarBlue^®^ reduction.

#### 2.6.2. Immunofluorescence Staining

To observe the osteogenic markers, bone sialoprotein (BSP) and osteocalcin, immunofluorescence staining was performed. After 21 days of cell culture, the samples seeded with 4 × 10^3^ human MG63 were fixed with 4% PFA/PBS during 10 min at 4 °C, then permeabilized with 0.1% Triton X-100 for 1 h, and incubated for 30 min with Alexa Fluor 546-conjugated phalloidin (Molecular Probes; Life Technologies, Fisher Scientific, Illkirch, France) for F-actin labelling and 5 min with 200 nM DAPI (Sigma-Aldrich Co., Darmstadt, Germany) for nuclear staining. Bone growth induction was measured by assaying expression of osteocalcin and Bone Sialoprotein-BSPII, using polyclonal goat anti-osteocalcin (1/200; Santa Cruz Biotechnology Inc., Heidelberg, Germany), and monoclonal mouse anti-BSPII (1/200; Santa Cruz Biotechnology Inc.) overnight at 4 °C. After rinsing with PBS, the cells were incubated with secondary antibodies; anti-goat Alexa fluor 488 diluted to 1/200 for osteocalcin and anti-mouse Alexa fluor 488 diluted to 1/200 for BSPII. The samples were observed under an epifluorescence microscope (LEICA DM 4000 B).

## 3. Results and Discussion

### 3.1. Mechanical and Thermal Properties of Micro-Sized PLA Fibers

The mechanical and thermal properties of the three different filaments are described in the table in Figure 2. The average diameter, tenacity at break (cN/tex), Young modulus (MPa), and crystallinity (%) were determined from DSC curves (see Supplementary Materials). The glass transition temperature (60 °C) and melting temperature (165 °C) determined from the DSC curves were nearly the same for the three filaments. However, the degree of crystallinity calculated from the respective DSC curve of each filament was different for the three filaments. In fact, drawing leads to an orientation of the PLA polymer chains, which increases the degree of crystallinity for the finest filament [47]. Concerning, the mechanical properties, the Young Modulus and tenacity values are similar for the 100 µm and 230 µm filaments but are also half of those of the 30 µm filament. For the 30 µm filament, the higher speeds used for Rolls 1 and 2 in the spinning machine (Figure 2) led to greater drawing of PLA filaments and to a higher orientation of polymer chains leading to the highest tenacity. Though the mechanical properties of the 100 µm-PLA and 230 µm-PLA were lower, they were high enough to allow these filaments to resist the mechanical constraints during the weaving process used.

**Figure 4:**
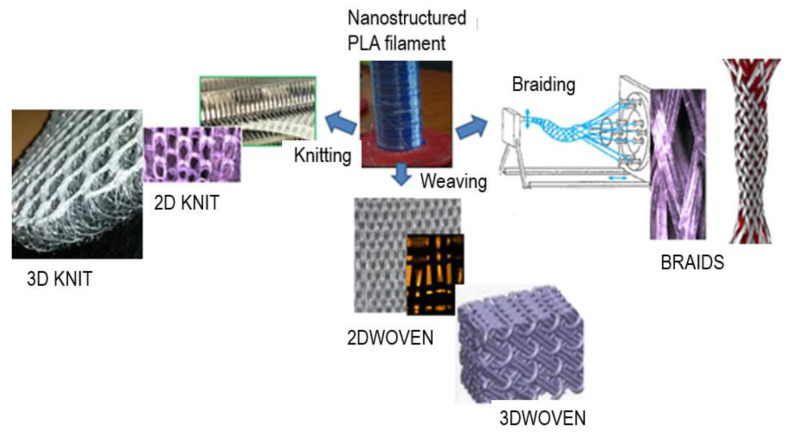
Processing ability of 100 µm and 230 µm diameter PLLA filaments into different 2D-3D structures (by weaving, knitting, and braiding). Minimum pore sizes ranged from 250–400 µm for the knitted scaffold, and from 50–100 µm for the woven, and 250–400 µm for the braids.

Indeed, the different textile processing methods (Figure 4) used showed that the 100 µm and 230 µm filaments could be processed into different 2D and 3D woven, knitted, and braided scaffolds, using onsite weaving, knitting, and braiding machines. Minimum pore sizes ranged from 250–400 µm for the knitted scaffold, and from 50–100 µm for the woven, and 250–400 µm for the braids.

### 3.2. Topographical Analysis of PLA Filament Surface Analyzed by Atomic Force Microscopy

**Figure 5:**
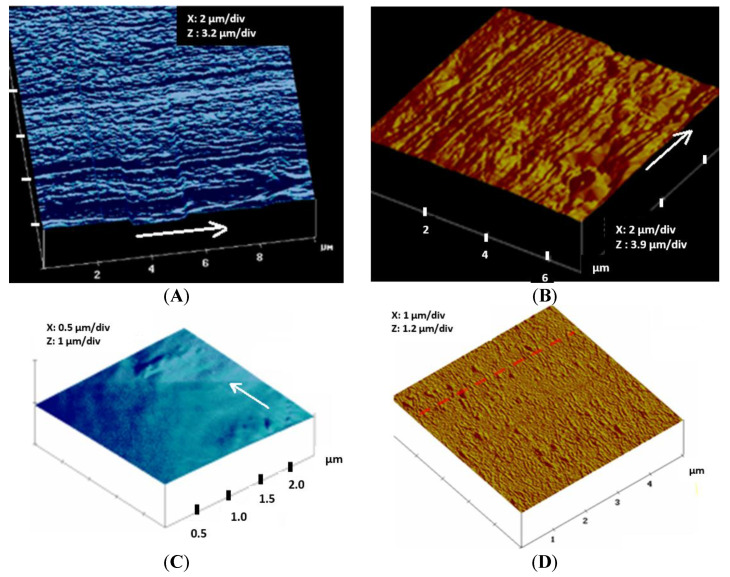
Typical topographical image obtained by Atomic Force Microscopy (in Tapping Mode) of PLLA filaments of diameter 30, 100 and 230 µm compared to a PLA film. White arrows indicate fiber length direction. (**A**) PLLA fiber (diamter100 µm); Ra = 80 nm; Rq = 120 nm; (**B**) PLLA fiber (diameter 230 µm); Ra = 52 nm; Rq = 70 nm; (**C**) PLLA fiber (30 µm); Rq = 3 nm; Ra = 6 nm; (**D**) PLLA film; Rq = 13 nm; Ra = 2 nm.

Figure 5A–C shows, respectively, typical topographical images obtained by Atomic Force Microscopy for each of the three different filaments compared to an extruded PLA film (Figure 5D), which was also subjected to biological tests.

While a nearly very smooth surface was obtained for the 30 µm microfilament, the 100 µm and 230 µm diameter microfilaments have similar surface patterns in the form of irregular parallel ridges which extend through the whole filament length, as already observed by Cicero et al. [45]. In the case of the 100 µm filament, the surface ridges are thinner, and the number of ridges/µm is higher than that on the 230 µm fiber surface. Much broader ridges as well as some unevenly distributed bumps appear on the 230 µm diameter filament surface. The average roughness parameters, R_a_ and R_q_, for the 100 µm diameter filament, were R_a_ = 80 nm and R_q_ = 120 nm, while for the 230 µm diameter filament, R_a_ = 52 nm and R_q_ = 70 nm, respectively.

### 3.3. Properties of Woven and Nonwoven PLA Scaffolds

Morphological analysis by photography and binocular lenses (Figure 3B) show the structure of the two woven scaffolds (WovenPLA100 and WovenPLA230, respectively, made from the 100 µm and 230 µm diameter filaments), and of the nonwoven scaffold NWPLA30 (made from the 30 µm diameter fibers). Table 1 summarizes the characteristics of these fibrous scaffolds: thickness, areal density, water contact angle, average pore size, and porosity of each textile scaffold.

Porosity (varying from 70 to 90%), as well as pore sizes (between 50 and 230 µm) obtained, were in the order of those specified in the literature [27,28] for the scaffolds used in Bone Tissue Engineering.

Wettability measurements carried out on the different textile structures using the tensiometry method describe in Section 2.5.1 allowed the calculation of the average water contact angles (WCA), which were 130°, 83°, and 76°, for the 30 µm, 100 µm, and 230 µm diameter PLA filaments, respectively. The WCA for PLA reported in literature is approximately 80°. The higher water contact angle of the nonwoven can be explained by the micro-roughness of the nonwoven surface due to the randomly arranged 30 µm-sized fibers at the nonwoven surface (see Figure 3-NWPLA30). Surface roughness due to ridges on the 100 and 230 µm diameter filaments seems to have little influence on the water contact angle measured.

### 3.4. In Vitro Colonization and Osteogenic Expression Induced by the PLLA Scaffolds

#### 3.4.1. Cell Proliferation Assay

##### Cell Adhesion and Proliferation after 3 and 6 Days for Cell Seeding over the PLA Discs

**Figure 6:**
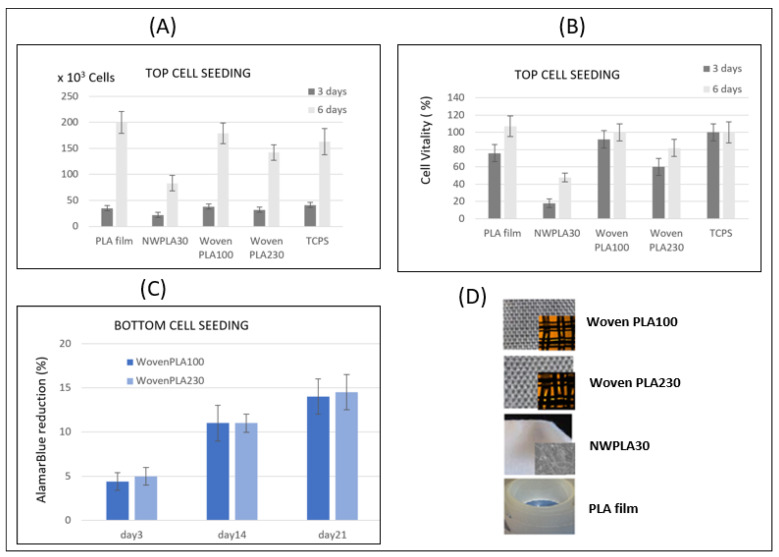
MG 63 osteoblast cell growth on different PLLA scaffolds. (**A**,**B**), after 3 and 6 days of culture for cell seeding with 6 × 10^3^ cells carried out on top of scaffolds expressed as the number of adhering cells—(**A**) and as Cell vitality (using AlamarBlue^®^) with respect to TCPS controls (100%)—(**B**); (**C**) after 3, 14, and 21 days of culture for cell seeding with 4 × 10^3^ cells carried out under the woven PLLA samples: cell growth and proliferation were assessed by AlamarBlue^®^ reduction; (**D**) Pictures of the PLLA scaffolds tested- two woven scaffolds WovenPLA100 and WovenPLA230 made from nanostructured PLLA filaments, and a nonwoven scaffold NWPLA made from a smooth 30 µm diameter filament, and a PLLA film.

Seeding of the Human MG 63 cells was carried out onto the scaffolds already placed in the 24-well cell culture plate. Figure 6A shows the number of MG 63 pre-osteoblastic cells counted after 3 and 6 days of culture, for an initial seeding with 6 × 10^3^ cells. Cell proliferation assays show that the number of cells adhering on the woven PLA scaffolds is multiplied by 6, after 3 days of culture and, after 6 days of culture, there are more than 23 to 30 times the number of cells adhering, compared to day 0. Among the two woven substrates, the WovenPLA100 leads to better cell adhesion and proliferation. Indeed, cell proliferation seems to be even higher on the WovenPLA100 (~180 × 10^3^ cells) compared to the tissue culture polystyrene TCPS (~165 × 10^3^ cells), which is the most commonly used film surface in mammalian cell culture.

Figure 6B presents the intensity of fluorescence due to the “resazurin reduction” of AlamarBlue^®^ molecules corresponding to the cellular metabolic activity (cell vitality), as compared to the standardized 100% fluorescence of cell cultured on TCPS membrane. It shows that the WovenPLA100 induced a near 90% cell activity (compared to 60% for the WovenPLA230) after 3-days culture, increasing to more than 100% after 6 days, indicating an excellent cell function. The MG 63 cells can also adhere and proliferate in the nonwoven PLA (NWPLA30) composed of 30 µm diameter PLA fiber. However, the number of cells adhering after 3 days and 6 days, respectively, is half of that on the WovenPLA100 for the same culture duration. Cell vitality on NWPLA30 reaches only 50% with respect to that on the TCPS, after 6 days. This is most probably due to the influence of the fiber diameter (30 µm) on the bone cell adhesion, as described in several published papers [31]. According to the results, the 100 µm diameter filament seems to be the best for promoting MG63 adhesion and proliferation.

##### Cell Adhesion and Proliferation Assay Using AlamarBlue^®^ up to 21 Days for Cell Seeding under the PLA Wovens

Only the two woven PLA scaffolds were subjected to this assay. Each woven PLA disc was placed in a 48-well cell culture plate already seeded with 4000 MG 63 osteoblast cells.

The AlamarBlue^®^ reduction percentage, followed over time at 3, 14, and 21 days (Figure 6C), confirmed the viability of the cells on both types of scaffolds (WovenPLA100 or WovenPLA230). However, cell colonization seems to be similar for both woven scaffolds. The results do not reveal any significantly enhanced effect of the fiber diameter. From these results, it becomes clear that, even after a longer period of time, both woven PLA scaffolds remain non-cytotoxic for human MG63, and that these cells continue to proliferate.

#### 3.4.2. Immunofluorescence Staining In Vitro Osteogenic Expression Induced by the Two Woven PLA Scaffolds

To observe the cells adhering on the PLA woven filaments as well as osteogenic markers bone sialoprotein (BSP) and OCN (osteocalcin), immunofluorescence staining was performed after 21 days of culture.

**Figure 7:**
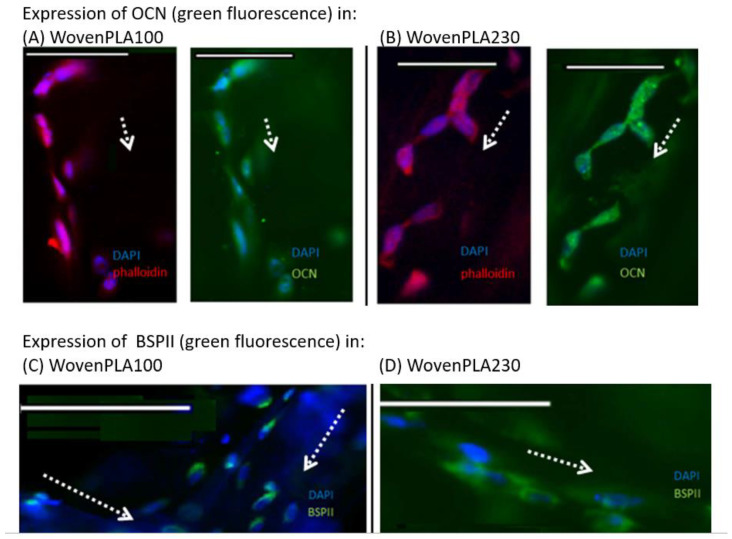
In vitro expression of OCN and BSPII in MG 63 cells, after 21 days of cell culture. (**A**,**B**) show expression of OCN in MG 63 cells adhered onto WovenPLA100 and WovenPLA230, respectively. The nuclei were stained with DAPI (in blue), actin labelled with phalloidin (in red), and OCN stained in green; (**C**,**D**) show expression of BSPII in MG 63 adhered onto WovenPLA100 and WovenPLA230. The nuclei were stained with DAPI (in blue), BSPII were stained in green. (For all images, white scale bars = 100 μm. White dotted arrows indicate filament length direction).

The staining of cells for nuclei (DAPI-in blue) and actin filaments (phalloidin-in red) showed that the osteoblasts adhered onto the scaffold in clusters along the fiber length axis for both wovens (Figure 7A,B). Larger and far more spread out and elongated cells anchored onto the WovenPLA230 through numerous filopodia extensions, which represent a typical morphology for this kind of cell. The staining of cells for nuclei (DAPI-in blue) and of osteocalcin in green (Figure 7A,B) shows that the expression of osteocalcin is significant.

Figure 7C,D show that protein expression of BSPII is significant in vitro, and that osteogenesis occurs successfully in both types of scaffold. However, a higher cell nuclei density can be observed on the WovenPLA100.

##### In Vitro Osteogenic Expression Induced Woven PLA Scaffolds

Expression of BSPII and osteocalcin occurred at an early stage on both scaffolds in the in vitro model. OCN is a bone protein synthesized primarily by osteoblasts and is an important marker of osteoblast differentiation. This protein participates in osteoblast activation to enable bone remodeling and regeneration. It also has an affinity for hydroxyapatite. Therefore, the expression of OCN highlights the mineralization phase in the process of bone remodeling.

Expression of BSPII shows that ECM is synthesized and that the mineralization phase has started. As both OCN and BSPII proteins are expressed, it would mean that both of the woven PLA scaffolds (WovenPLA230 and WovenPLA100) would be suitable for the field of bone regeneration.

## 4. Final Discussion

PLA, and especially PLLA, is a biodegradable polymer used as biomaterials for orthopedic fixation, ligaments, and tendons [50]. Nanofibers of this polymer show the promotion of the growth of cells, such as osteoblast cells [51,52].

For use as scaffolds in Bone Cell Engineering, the surface properties including surface topography and surface energy, as well as pore size, are important parameters. As microfilaments can provide various scaffolds with tunable size pores suitable for cell infiltration inside the scaffold, this study has been focused on a simple one-step fiber melt-spinning method to add nano-structuration to micro-sized PLA filaments having a diameter of 100 µm and 230 µm. Spinning conditions can be modified by varying speeds at the exit of spinning die and that of the fiber drawing step. Thus, ridge-shaped nanostructures were produced on both 100 and 230 µm diameter PLA fibers, running in the direction of the fiber length, as confirmed by Atomic Force Microscopy. A higher periodicity of ridges is observed in the case of the 100 µm fiber compared to the 230 µm diameter fiber. Indeed, the ridge-shaped nanostructures on the biodegradable PLA fibers produced in this study would provide ideal surface topography for osteoblast cell adhesion.

The appearance of such ridge-shaped structures on the PLA fiber surface, oriented in the direction of fiber length, was attributed to the presence of nanofibrils on the outer core of the fiber (Cicero, 2002). This phenomenon has been explained by the flexible molecular backbones of the PLLA polymer [45]. In the literature, nanofibrils of diameter 30 to 60 nm were observed. Within each microfibril, the alternating crystalline and amorphous regions are stacked, whereas the interfibrillar area is presumed to be populated by extended but uncrystallized molecules.

In the case of the 100 µm fiber, nanofibrils of 200 nm (pale shade ridges) separated by inter-fibril distances of about 200–400 nm are observed, while larger nanofibrils (200–400 nm) are present on the 230 µm diameter PLA fiber. Most probably, the lower drawing out speed of Roll 2 (Figure 1) during melt–spinning would not allow enough formation of the thinner nanofibrils on the 230 µm PLA fiber. Average roughness parameters for the 100 µm diameter fibers, were R_a_ = 80 nm and R_q_ = 120 nm, while for the 230 µm diameter PLA, R_a_ = 52 nm and R_q_ = 70 nm, respectively.

Several researchers [5] have shown that surfaces of roughness in tens of nanometers are preferred by bone cells for adhesion, growth, differentiation, and phenotypic maturation, rather than flat surfaces, and surfaces with submicron- or micro-scale roughness [6,14,16,21]. Moreover, alignment of cells along grooves, steps and ridges have been reported. Lenhert et al. [23] showed that grooves of 50–150 nm depth and with periodicity of 500 nm promote the alignment of osteoblasts along the grooves. Indeed, the high ridge periodicity produced on the biodegradable100 µm and 230 µm PLA fiber surfaces seems to favor osteoblast cell adhesion. Though the PLA film provides an appropriate surface for cell adhesion and proliferation, it is a non-porous 2D structure, and cannot produce porous 2D/3D scaffold structures that can be fabricated using the two different filaments. Such porous structures allow nutrient diffusion and cell migration.

The nanostructured microfibers have reduced mechanical tensile strength compared to smaller-diameter fibers (diameter 10 to 50 µm) used in textiles, because the lower draw ratio used for their fabrication leads to a reduction in crystallinity content. Nevertheless, such nanostructured microfibers can still be processed into 2D-3D textile scaffolds, such as wovens but also braids and knits (Figure 4). In this work, porous woven scaffolds, made from each of the microfilaments and with pore sizes between 50 and 100 µm, were used for biological testing.

Biological tests were carried out by seeding osteoblastic MG 63 cells either underneath or over each woven scaffold. Whatever the case, both cell counting and AlamarBlue^®^ tests showed that the two woven scaffolds enable in vitro colonization. In vitro osteogenic expression was induced on both wovens, as shown by the expression of osteocalcin and bone sialoprotein (BSPII), 21 days after seeding, at an early stage. OCN is a bone protein synthesized primarily by osteoblasts and is an important marker of osteoblast differentiation. This protein participates in osteoblast activation to enable bone remodeling and regeneration. It also has an affinity for hydroxyapatite. Therefore, the expression of OCN highlights the mineralization phase in the process of bone remodeling. Expression of BSPII shows that ECM is synthesized and that the mineralization phase has started. As both OCN and BSPII proteins are expressed, it would mean that both of the nanostructured microfilaments, FiPLA100 and FiPLA230, would be suitable for the field of bone regeneration.

Though both woven PLA scaffolds would be suitable for the field of bone regeneration, the higher diameter of the fiber in the woven PLA 230 would allow increased cell spreading of the osteoblast on the fiber surface, as confirmed by the elongated filopodia intimately anchored to the nano-structured 230 diameter filaments. Thus, we report a new generation of biodegradable nano-structured microfiber which can be processed into fibrous scaffolds of varying 2D, 3D shapes, and tunable pore-sizes and porosities.

## 5. Conclusions

Nanostructured and living biomaterials are innovative technologies which improve the quality of life and could reduce costs. We report for the first time a single-step fabrication of nano-structured microfilaments for use as scaffolds in Bone Tissue Engineering. Melt-spinning is not, on its own, an innovative process. However, monitoring of spinning conditions allows the production of, in a one-step process, microfibers with nanoscale aligned ridges, with appropriate diameter suited for bone cell adhesion and proliferation. The two woven scaffolds made from the nano-structured microfibers enabled in vitro colonization, and induced in vitro osteogenic expression, as shown by the expression of osteocalcin and bone sialoprotein (BSPII), 21 days after seeding. The results are very interesting since the perspective of forming various 3-D structures suited for various applications, including large bone lesions, using the nanostructured micro-sized PLA fibers is tremendous.

Further experiments should be carried out to assess in vivo mineralization. Ideally, scaffolds should also be equipped with bioactive molecules, including growth factors, or drugs to prevent adverse biological response, such as sepsis or cancer recurrence. So, the functionalization of the woven scaffolds by active materials, such as growth factors (BMP-7) and drugs, should also be tested. Finally, the adhesion and proliferation of other types of cells such as fibroblasts, epithelial, and macrophage cells, which are influenced by nanometrically deep features, can be tested on this new generation of biodegradable nano-structured microfiber scaffolds. In addition, different polymers such as PCL or a combination of PCL/PLA, could be used to produce the nano-structured microfibers by the melt–spinning, to obtain tunable rate of scaffold properties (degradation, surface energy, etc.). This study is the starting point for future research on the fabrication and use of a new generation of 3D porous fibrous biomaterials.

## Figures and Tables

**Table 1:** Properties of PLA scaffolds (wovens and nonwoven).

Textile Scaffold	Fiber Diameter (μm)	Water Contact Angle (°)	Areal Density (g/m^2^)	Thickness (μm)	Porosity (%)	Average Pore Size and Morphology (μm)
Woven PLA100	100 ± 5	83 ± 2°	144 ± 5	590 ± 5	80 ± 2	Rectangular pore 100 × 100 µm
Woven PLA230	230 ± 10	76.0 ± 3°	273 ± 5	790 ± 5	72 ± 4	Rectangular pore 50 × 100 µm
Nonwoven NWPLA30	30 ± 3	130 ± 5°	265 ± 4	1000 ± 10	90 ± 3	undefined pore morphology

## Data Availability

Not applicable.

## Supplementary Materials

The following supporting information can be downloaded at: https://www.mdpi.com/article/10.3390/molecules27123790/s1, Figure S1: Thermal property of micro-sized 230 µm diameter PLA fiber as measured by Differential Scanning Calorimetry (DSC), which allows to determine the crystallinity rate of PLA monofilaments. Figure S2: Stress/strain diagram for the PLA30.

## Sample Availability

Samples of the compounds are available from the authors.
