# Supplementary material for: Nano-Structured Ridged Micro-Filaments (≥100 µm Diameter) Produced Using a Single Step Strategy for Improved Bone Cell Adhesion and Proliferation in Textile Scaffolds
Source: Molecules. 2022 Jun 13;27(12):3790. doi: 10.3390/molecules27123790 (PMC9228432; doi:10.3390/molecules27123790)

Supplementary information :

Figure S1 : Thermal property of micro-sized 230µm diameter PLA fiber as measured by Differential Scanning Calorimetry (DSC), which allows to determine the crystallinity rate of PLA monofilaments.

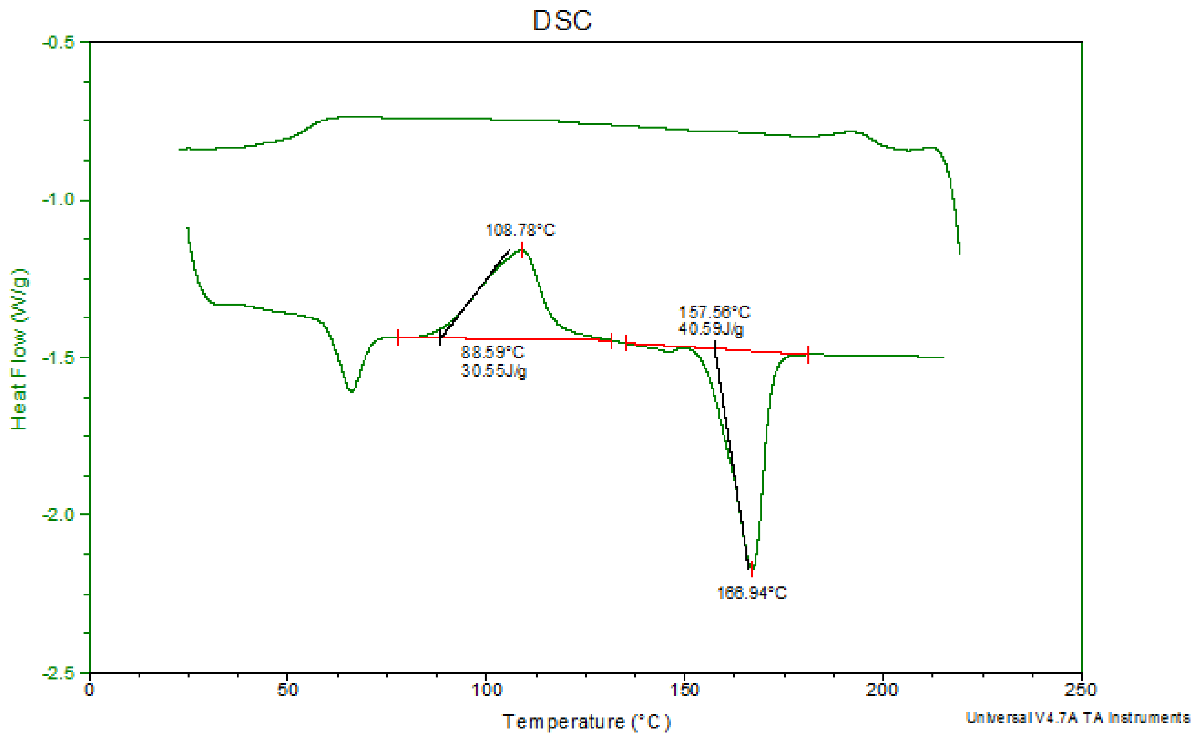

Figure S2: Stress/strain diagram for the PLA30

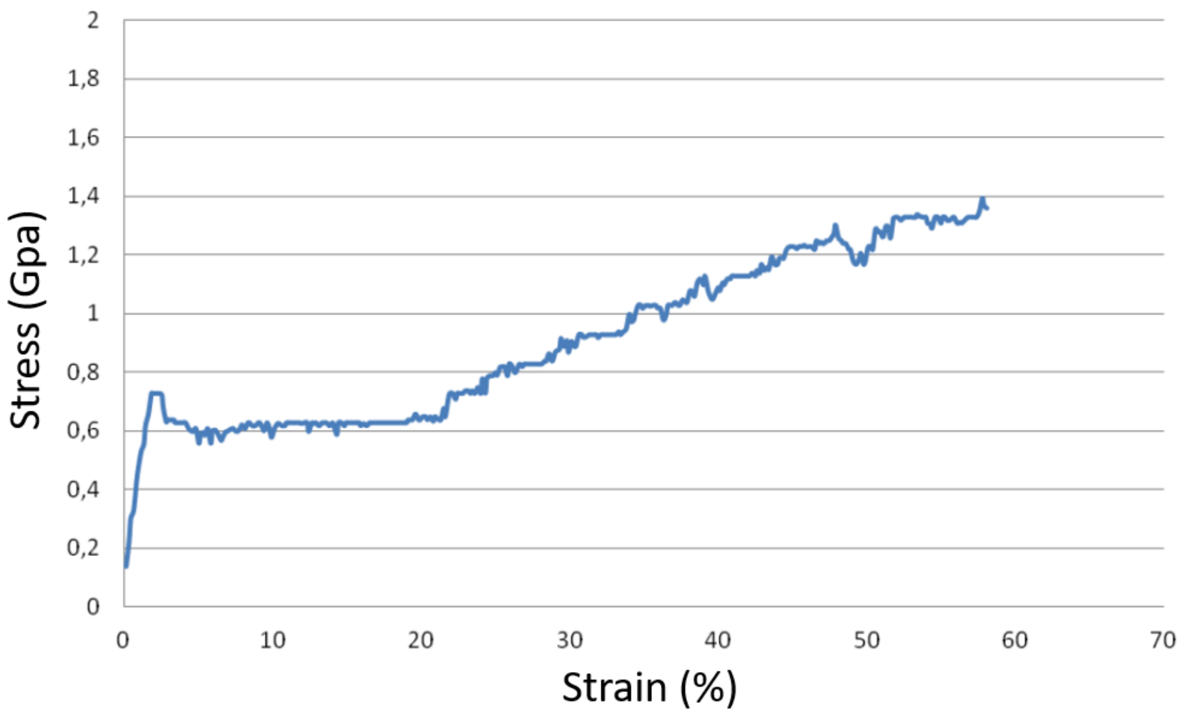

Supplement: Supplementary file 1 [file molecules-27-03790-s001.zip › molecules-1697092-supplementary.pdf]
